# Quality of Diabetes and Hypertension Management at the DAWN (Dedicated to Aurora’s Wellness and Needs) Student-Run Free Clinic

**DOI:** 10.7759/cureus.9539

**Published:** 2020-08-03

**Authors:** Caitlin Felder-Heim, Kari Mader

**Affiliations:** 1 Family Medicine, University of California San Francisco, San Francisco, USA; 2 Family Medicine, University of Colorado School of Medicine, Aurora, USA

**Keywords:** student-run clinic, student run free clinic, health care outcomes, diabetes, hypertension, quality improvement, unequal access to health care, health care disparity, interprofessional care, chronic disease management

## Abstract

Introduction

Student-Run Free Clinics (SRFCs) are part of the safety-net healthcare system. Given variable settings and models, relatively little is known about the quality of care in these settings.

Methods

A mixed-methods evaluation of diabetes and hypertension management was conducted for patients initiating care from March 1, 2015, to September 31, 2016, at the DAWN (Dedicated to Aurora’s Wellness and Needs) SRFC. Retrospective chart review assessed whether patients received recommended screening tests (process outcomes) and achieved disease control (short-term outcomes). These outcomes were compared to a local community health center (CHC), a local federally qualified health center (FQHC) network, and Colorado Medicaid (CoM) using one proportion t-tests. In-depth case studies of randomly selected individuals with good and poor disease control identified targets for quality improvement through nominal group technique.

Results

Diabetic patients (n=30) were recommended screening, including HbA1c (93.3%) (vs. 77.8% with CoM, p=0.04), nephropathy care (70%) (vs. 85.4% with CoM, p=0.02), retinopathy examination (30%) (vs. 40.47% with CoM, p=0.24). Diabetic short-term outcomes showed 46.6% with poor control (vs. 61.1% at the CHC, p=0.10; vs. 30.62% at the FQHC, p=0.06; vs. 55% with CoM, p=0.10). Patients with hypertension (n=75) 33.3% had controlled (<140/90) blood pressure (vs. 49.2% at the CHC, p<0.01; vs. 61.1% at the FQHC, p<0.01; vs. 58.9% with CoM, p<0.01). Themes for quality improvement included improving follow-up, documentation and data collection, clinic processes, and addressing barriers to care.

Discussion

DAWN outcomes were comparable to other safety-net providers for diabetes, similar to findings in evaluations conducted by other SRFCs. However, DAWN did not have equivalent outcomes for hypertension in contrast to other published findings from SRFCs. Poor access to care and baseline chronic disease control among DAWN patients may have contributed to these findings.

Conclusions

While this study is not directly generalizable to all SRFC models and communities, these results contribute to the growing body of data around SRFCs and chronic disease management and indicate that SRFCs may have a role in the safety-net healthcare system. However, more study is needed to ensure that SRFCs can provide high-quality care because otherwise efforts should focus on other strategies to expand access within the safety-net system.

## Introduction

The safety-net healthcare system represents a key intervention for increasing access to care among uninsured patients and thereby mitigating the impacts of chronic disease and improving health outcomes [1]. However, the existing safety-net system is often unable to accommodate all patients who need care [2]. In this setting, student-run free clinics (SRFCs) can be an important source of care for uninsured patients, in addition to representing a learning opportunity for health professional students. SRFCs are present in at least 75% of U.S. medical schools and operate under a variety of different models, though universally they have limited hours and resources compared to full-time clinics [3]. Given the prevalence of SRFCs and diversity of clinic models, it is imperative that SRFCs evaluate patient care outcomes to ensure that they are comparable to clinics where uninsured patients typically access care and to identify opportunities for quality improvement [4,5]. Evaluations at SRFCs have examined the quality of chronic disease management, quality of preventative care, and patient satisfaction [6-9]. The DAWN (Dedicated to Aurora’s Wellness and Needs) clinic, as a new SRFC, felt that it was important to engage in an evaluation of quality of care.

DAWN is an SRFC in Aurora, Colorado, USA, founded with support from a community-based non-profit partner and the University of Colorado Anschutz Medical Campus. At DAWN, interprofessional teams of students and licensed providers bring primary care to uninsured adult patients in Aurora [10]. The clinic is open one evening per week for comprehensive primary care services including general medical, dental, pharmacy, physical therapy, psychology, and care coordination. The clinic is open two additional nights per week for physical therapy and specialty services. While DAWN has improved access to care for at least some uninsured patients in Aurora, DAWN should ensure that care meets national quality standards at similar rates to other safety-net providers, otherwise, it cannot be considered an effective intervention for controlling chronic disease and should initiate quality improvement efforts. The purpose of this study was to (1) understand DAWN’s ability to achieve quality-of-care performance standards for diabetes and hypertension similar to other safety-net providers and (2) to identify quality improvement targets that may lead to improved chronic disease management.

## Materials and methods

This project was exempt from human subjects research under COMIRB Protocol 17-1152 as a quality improvement and program evaluation project. The primary method was a quantitative chart review to determine diabetes and hypertension process and short-term outcome measures for DAWN patients initiating care between March 1, 2015, and September 31, 2016, and to compare those measures to nearby safety-net clinics and Colorado Medicaid (CoM) data. The time frame was chosen as it represented all of the data available at the time of the evaluation and using a shorter time frame would have decreased the sample size. An evaluation team of 12 trained students completed the chart review. Study data were collected and managed using the Research Electronic Data Capture (REDCap) tool hosted at the University of Colorado, Denver [11]. Aggregate data were exported for analysis and stored on a secure HIPAA-compliant online server.

The comprehensive retrospective chart review included all non-pregnant adult patients (age 18 years or older) with a diagnosis of diabetes or hypertension. Exclusion criteria included pregnant women and any patients with an outside primary care provider within three months of initial contact at DAWN. Eligible patients were identified by diagnosis codes and all further data were collected manually by the evaluation team and entered into REDCap. Data collected included background data (age, sex, number of visits), and process and short-term outcome measures based on national guidelines at the time of the evaluation from the Healthcare Effectiveness Data and Information Set (HEDIS), the American Diabetes Association (ADA), and the Joint National Committee (JNC-7) [12-14]. The diabetes process outcomes included a yearly HbA1c screen, nephropathy screen (or ACE-inhibitor prescription), retinopathy screen, a one-time lipid panel, and prescription of at least one anti-hyperglycemic agent [12,13]. Neuropathy screening proved too difficult to extract from charts. Short-term diabetic outcomes included good (<8.0) and poorly controlled (>9.0) HbA1c level [12,15].

The following process outcomes for hypertension were collected: blood pressure measurement with each visit, prescription of a single blood pressure medication for stage 1 hypertension (140-159/90-99 mmHg), or prescription of two blood pressure medications for stage 2 hypertension (160/100 mmHg) [12,14]. The short-term outcome measure for hypertension was adequate blood pressure control (<140/90 mmHg) at the last appointment during the study period [12]. If any process or outcome measures were missing, they were assumed to be poorly controlled or not performed; this was the same way these data were collected for the comparison groups.

Data for comparison to DAWN were drawn from published outcome data from the Health Resources and Services Administration (HRSA) and CoM [15-18]. Clinics appropriate for comparison included a community health center (CHC) in the city of Denver and a federally qualified health center (FQHC) network spanning both Aurora and Denver. These clinics were chosen because they serve a similar patient population and geographic area. Individuals insured by CoM were used as a third comparison group as these patients are often cared for in CHCs or FQHCs, and there were additional process and outcome data for this population that was not available from the other comparison safety-net clinics. Furthermore, Medicaid data have been used for comparison in similar studies of other SRFCs [8,19].

Data were analyzed using online statistical calculators and SPSS statistical software (IBM Corp., Armonk, NY, USA) [20,21]. Where available, DAWN’s achievement of HEDIS process and short-term outcome measures for diabetes and hypertension were compared to CoM and CHC/FQHC outcomes using tests for one proportion. Tests for one proportion were used because the drastic differences in population size between CoM (>35,000), CHC (>4,500), FQHC network (>50,000), and DAWN (<100) meant that comparisons using two-proportion tests would be less meaningful. DAWN’s results were analyzed as observed proportions based on sample size and compared to the CHC, FQHC network, or CoM outcomes as a standard null hypothesis.

The primary study was paired with in-depth case studies using chart review to identify unique barriers or variations in care that may have impacted disease control. A random sample of patients with both well-controlled and poorly controlled diabetes (n=10) and hypertension (n=20) were selected. In-depth chart review was quantitative and qualitative in nature. Comprehensive demographic data, social determinant of health screening results, and the presence or absence of certain DAWN processes were examined. Examples of data collected included whether the chart indicated medication adherence issues, number of visits, whether appropriate pre- and post-visit communication occurred, presence of care coordination involvement in visits, and composition of the care team (e.g. number and specialty of students and faculty). Statistical analyses were not performed due to the small sample size. This data collection was summarized and reviewed for both well-controlled and poorly controlled groups to identify trends and to guide the nominal group technique (NGT) discussion. NGT is a method for conducting group brainstorming and prioritization of ideas or problems [22]. Evaluation team members reviewed identified trends and utilized NGT to brainstorm and prioritize quality improvement targets [23].

## Results

A total of 30 patients at DAWN met inclusion criteria and were included in the diabetes cohort and 75 patients met criteria for the hypertension cohort. Patients who had both hypertension and diabetes (n=21) were included as independent data points in both groups. Demographic data available for comparison groups and DAWN patients included in this study are reflected in Tables 1, 2. DAWN had a significantly higher elderly (age > 65) population compared to the CHC and FQHC network. DAWN had a significantly higher percentage of patients who identify as Hispanic/Latino compared to CoM and the FQHC network but significantly less than the CHC. Compared to CoM, DAWN had a significantly higher prevalence of patients who rated their health as fair or poor and who reported not having a usual source of care. DAWN also had the highest rate of uninsured patients among the comparison groups.

**Table 1:** Clinic/Patient Demographics *p<0.05 (respective clinic compared to DAWN). **p<0.001 (respective clinic compared to DAWN). Ꝋ Data unavailable. #Defined by DAWN as patients who reported having a primary care provider. DAWN, Dedicated to Aurora’s Wellness and Needs; FQHC, federally qualified health center

	Colorado Medicaid		FQHC Network		Community Health Center		DAWN (All)	
Variable	%	N	%	N	%	N	%	N
Age (adults)								
Age: 18-64 years	81.5*	265516	92.4*	31905	95.6**	4292	88.3	280
Age: 65+ years	18.5**	60423	7.6%*	2620	4.4**	197	10.7	34
Sex								
Males	42.3	318640	40.5	Ꝋ	40.5	Ꝋ	45.52	132
Females	57.7	434750	59.5	Ꝋ	59.5	Ꝋ	54.48	158
Ethnicity								
American Indian/Alaska native	1.2	8756	0.5	273	2.2	105	1.40	3
Asian	2.0	15234	6.2*	3128	3.3	157	1.86	4
Black/African American	6.8**	51438	12.5*	6331	13.1*	630	18.14	39
Hispanic/Latino	33.3**	250731	50.2**	25363)	96.5**	4634	70.23	151
Native Hawaiian/Pacific Islander	0.4**	3283	0.4**	188	0.5*	26	1.86	4
White	36.0**	271106	28.5**	14384	1.9**	93	6.51	14
Insurance status								
Yes	100	Ꝋ	59.61**	30106	12.10**	581	0	Ꝋ
No	0	Ꝋ	40.39**	20396	87.90**	4221	100	Ꝋ
Self-reported health								
Poor or fair	25.9**	Ꝋ	Ꝋ	Ꝋ	Ꝋ	Ꝋ	54.40	266
Usual source of care^#^								
Yes	84.0**	Ꝋ	Ꝋ	Ꝋ	Ꝋ	Ꝋ	8.91	22
No	16.0**	Ꝋ	Ꝋ	Ꝋ	Ꝋ	Ꝋ	91.09	225

**Table 2:** DAWN DM and HTN Cohort Demographics DAWN, Dedicated to Aurora’s Wellness and Needs; HTN, hypertension; DM, diabetes mellitus

	DAWN Clinic (HTN)		DAWN Clinic (DM)	
Variable	Percentage	N	Percentage	N
Age (adults), years				
19-44	22.7%	17	23.3%	7
45-64	56.0%	42	53.3%	16
65-74	13.3%	10	16.7%	5
75-84	5.3%	4	6.7%	2
85+	2.7%	2	0.0%	0
Sex				
Males	48.0%	36	40.0%	12
Females	52.0%	39	60.0%	18

**Figure 1:**
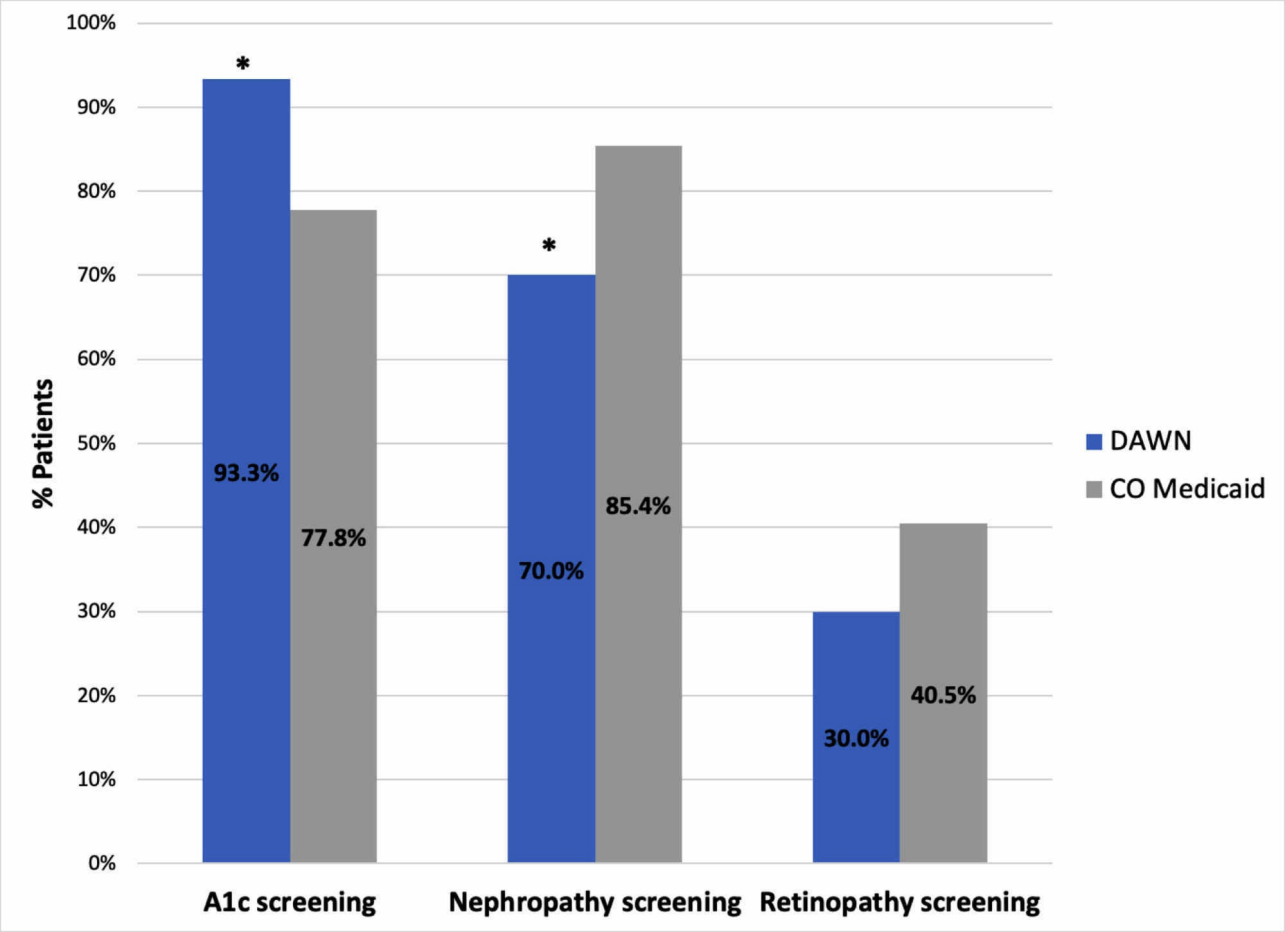
DAWN Diabetes Process Outcomes *p<0.05 DAWN, Dedicated to Aurora’s Wellness and Needs

**Figure 2:**
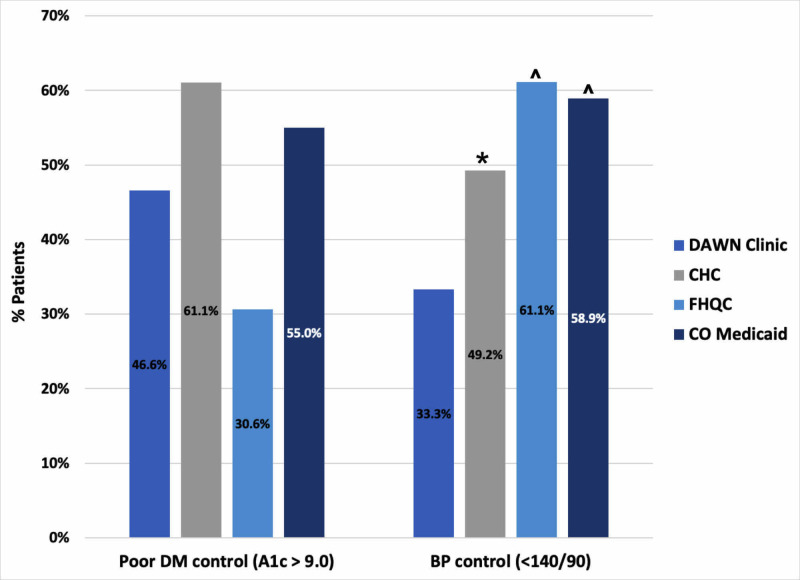
DAWN Diabetes and Hypertension Short-Term Outcomes *p<0.05 ^p<0.01 DAWN, Dedicated to Aurora’s Wellness and Needs; CHC, community health center; FQHC, local federally qualified health center; CO, Colorado; DM, diabetes mellitus; BP, blood pressure

The results of diabetes process outcomes are displayed in Figure 1. Compared to CoM, DAWN had significantly higher rates of HbA1c screening 93.3% (vs. 77.8%, p=0.04), lower rates of appropriate nephropathy care 70% (vs. 85.4%, p=0.02), and statistically equivalent rates of retinopathy screening 30% (vs. 40.47%, p=0.24). Data for diabetes process outcomes were not available from the local CHC and FQHC network. There were no significant differences in diabetes control at DAWN compared to the CoM cohort, CHC, and FQHC. Diabetic short-term outcomes showed 46.6% with poor control (vs. 61.1% at the CHC, p=0.10; vs. 30.62% at the FQHC, p=0.06; vs. 55% with CoM, p=0.10) (Figure 2).

DAWN had significantly lower rates of blood pressure control compared to all groups, with 33.3% having controlled (<140/90) blood pressure compared to 49.2% at the CHC (p<0.01), 61.1% at the FQHC (p<0.01) and 58.9% for those with CoM (p<0.01) (Figure 2). Process outcomes for hypertensive patients showed that 77.3% of patients received a blood pressure measurement at every visit (recorded in the appropriate place in the medical record) and 73.3% of patients were prescribed appropriate anti-hypertensive therapy. There were no data for comparison for hypertension process outcomes.

Quantitative trends revealed in the case studies showed poorly controlled patients were more likely to be male, had more clinical visits, and had more severe disease at baseline (higher HbA1c or higher blood pressure at baseline vs. well-controlled patients). Well-controlled diabetic and hypertensive patients had, on average, more visits that involved care coordination (3.13 vs. 2.33 visits with care coordination) and were more likely to have been contacted for follow-up after missing an appointment compared to poorly controlled patients (80.7% vs. 48.9%). Several cases revealed patients with inconsistent follow-up frequently returned with more poorly controlled disease. It was also observed that most patients in the case study indicated that they experienced significant barriers related to social determinants of health such as concerns about food, transportation, finances, and legal status. Lastly, the research team noted that documentation of care activities was frequently inconsistent in regard to both location in the health record and documentation completeness, thus assessing care activities was challenging and likely impacted the continuity of patient care.

The identified themes from the case review and results of the subsequent NGT process are noted in Table 3. The NGT process identified four thematic areas for improvement, including follow-up, clinic processes for chronic disease management, addressing barriers to care, and documentation and data collection.

**Table 3:** Nominal Group Technique Results

	Factor Description	Item Rank
Theme	Follow-Up	
	Identify patients with severe disease and target patients for retention	1
	Improve care coordinator follow-up of missed appointments	7
	Increase communication with patients (i.e., telephone follow-up)	10
Theme	Documentation/Data Collection	
	Increase consistency of documentation	2
	Improve completeness of patient intake/forms	6
	Harness medical record future quality-of-care analyses	9
Theme	Clinic Processes for Disease Management	
	Target nephropathy and retinopathy screening rates	4
	Increase health education for lifestyle changes for chronic disease management	5
	Establish clear goals for initial patient visit	8
Theme	Addressing Social Barriers To Care	
	Assess social determinants early on while creating a care plan	3
	Establish clear goals for social needs in initial patient visit	8

## Discussion

DAWN achieved quality standards at rates approximately equivalent to the comparison groups for diabetes but not for blood pressure. Comparing process measures for diabetic care to CoM, HbA1c screening rates were higher, nephropathy screening rates were lower, and retinopathy screening had no significant difference. HbA1c screening rates may have been higher because most patients initiated care at DAWN during the study period and an HbA1c would typically be drawn at that time. In contrast, clinics serving CoM patients would have needed to actively reengage some patients in care so that they might receive a yearly HbA1c screening. Nephropathy screening and management may have been lower due to lack of standard protocols for diabetes management, variations in practice between supervising attending clinicians, or medication access barriers. Notably, ophthalmology services were not introduced until mid-way through the study period, and if introduced sooner it is possible that retinopathy screening rates would have been higher. Diabetes-related short-term outcomes showed no significant difference between patients at DAWN and the comparison sites, suggesting comparable quality of diabetes management. Unfortunately, blood pressure control at DAWN was significantly lower than all comparison groups. This suggests that the quality of hypertension care in the first 18 months after DAWN opened may not have been as efficacious as clinics caring for similar populations.

These findings raise several considerations, especially in the context of the current literature. The limited available evidence around quality of care for patients with diabetes at SRFCs is concordant with our findings. Ryskina et al., Smith et al., and Gorrindo et al. found that SRFC achieved diabetes outcomes at rates comparable to or better than other providers or recommended by quality standards [8,19,24]. The diabetes outcomes at DAWN are impressive considering that the clinic was only in operation for 18 months at the time of this evaluation and is only open once per week with rotating volunteers. Additionally, more than 90% of patients coming to DAWN stated they had no prior access to primary care when establishing care at the clinic and thus likely had poor baseline diabetes control.

The finding that quality of hypertension care did not follow a similar pattern warrants discussion in regard to possible confounders, baseline control, and time of follow-up. Patients seeking care at SRFCs may have had poorer disease control at baseline than members of the comparison groups. While baseline disease control data were not available for comparison groups, DAWN patients reported significantly less access to primary care compared to the Medicaid comparison group (8.9% vs. 84.0%) and had lower baseline self-reported health scores (Table 1), which are both statistics correlated with overall health and mortality [25,26]. It is also possible that Colorado Medicaid and local CHCs had better hypertension outcomes because patients in those groups were engaged in care for longer than patients at the newly-established DAWN Clinic. Unfortunately, available comparison data did not allow for comparing time of follow-up. These confounders likely do not explain this finding in its entirety, as these characteristics would also likely apply to patients with diabetes, but diabetic outcomes were not worse than comparison groups.

The results for hypertension control differ from other studies on hypertension quality standards at other SRFCs. Smith et al., Wahle et al., and Zucker et al. all concluded that the SRFC evaluated was able to improve blood pressure and achieve national quality standards [27-29]. The current SRFC literature may be more favorable due to publication bias and the fact that only robust or long-standing SRFCs have the resources to conduct quality-of-care evaluations. Regardless, the findings compel DAWN to engage in quality improvement focused on hypertension control. The case studies that informed the NGT process provided important information for future quality improvement work.

This evaluation had several limitations. The study examines only a small population of patients followed for a relatively short period of time given DAWN's recent establishment and limited capacity. Lack of a well-matched control group and full datasets for comparison sites limited conclusions as noted. There are also likely unknown confounding factors that impact the ability to interpret differences between the DAWN clinic population and comparison groups. Due to the time-intensive nature of a manual chart review, a team of students was necessary to collect data and the team was not able to duplicate chart reviews and test inter-rater agreement. This increased the possibility of error in data collection, though these risks were mitigated through orientation and ongoing communication between evaluation team leaders and data collectors.

## Conclusions

Despite the limitations of the SRFC setting, DAWN was able to achieve approximately the same diabetes quality standards as other safety-net providers but was not able to achieve the same standards for hypertension control. DAWN still represents a promising intervention for improving access to care and reducing uncontrolled chronic disease in uninsured patients in Aurora. However, DAWN must carefully consider how to implement quality improvement and conduct further evaluations in order to be considered comparable to other safety-net providers. While this study is not directly generalizable to all SRFC models and communities, these results contribute to the growing body of data around SRFCs and chronic disease management and indicate that SRFCs may have a role in the safety-net healthcare system. However, more study is needed to ensure that SRFCs can provide high-quality care. If SRFCs do not meet quality standards, they must engage in quality improvement or focus on other strategies to expand access within the safety-net system.
